# OLT1177 (Dapansutrile), a Selective NLRP3 Inflammasome Inhibitor, Ameliorates Experimental Autoimmune Encephalomyelitis Pathogenesis

**DOI:** 10.3389/fimmu.2019.02578

**Published:** 2019-11-01

**Authors:** Alba Sánchez-Fernández, Damaris B. Skouras, Charles A. Dinarello, Rubèn López-Vales

**Affiliations:** ^1^Institut de Neurociencies and Departament de Biologia Cellular, Fisiologia i Immunologia, Universitat Autonoma de Barcelona, Bellaterra, Spain; ^2^Centro de Investigación Biomédica en Red de Enfermedades Neurodegenerativas, Madrid, Spain; ^3^Olatec Therapeutics LLC, NewYork, NY, United States; ^4^Department of Medicine, University of Colorado Denver, Aurora, CO, United States; ^5^Department of Medicine, Radboud University Medical Center, Nijmegen, Netherlands

**Keywords:** experimental autoimmune encephalomyelitis, OLT1177, NLRP3, inflammation, cytokines

## Abstract

IL-1β and IL-18 are pro-inflammatory cytokines that are linked to inflammation. Activation of the NOD-like receptor protein 3 (NLRP3) inflammasome is involved in the maturation and secretion of IL-1β and IL-18 and, thus, plays a key role in the pathogenesis of many inflammatory conditions, including multiple sclerosis (MS). OLT1177™ (Dapansutrile) is a newly developed drug that is safe in humans and inhibits specifically the NLRP3 inflammasome. In the present study, we investigated whether OLT1177 exerts therapeutic effects in experimental autoimmune encephalomyelitis (EAE), a mouse model of MS. We found that EAE mice fed an OLT1177-enriched diet prophylactically were significantly protected against functional deficits and demyelination in the spinal cord. We also demonstrated that prophylactic oral administration of OLT1177 led to marked reduction (~2- to 3-fold) in the protein levels of IL-1β and IL-18, as well as, IL-6 and TNFα, in the spinal cord of EAE mice. Moreover, prophylactic oral administration of OLT1177 significantly attenuated the infiltration of CD4 T cells and macrophages in the spinal cord. We also demonstrated that oral administration of OLT1177, starting at disease onset, resulted in significant amelioration of the clinical signs of EAE. Overall, these first data suggest that OLT1177 could have clinical benefit for the treatment of MS in humans.

## Introduction

Cytokines are critically involved in the course of a myriad of inflammatory diseases, such as MS (1). MS is a chronic, neuroinflammatory and demyelinating disease of the central nervous system (CNS) that affects around 2.5 million people worldwide. The etiology of MS is still poorly understood, but it is well-established that demyelination and further neurodegeneration are linked to neuroinflammation since infiltrated and activated resident immune cells are present in all stages of MS and in all patients (2–5).

Clinical studies have shown that progression of MS correlates with the dysregulation of some cytokine networks within the CNS (1, 6). IL-1β and IL-18 are two members of the IL-1 family with broad pro-inflammatory actions (7, 8) and both are significantly elevated in MS patients (9–12). Pharmacological blockade or genetic removal of IL-1β or IL-18 resulted in protection against the development of experimental autoimmune encephalomyelitis (EAE), the murine model of MS (13–16). Interestingly, it has been described that families that are characterized by high IL-1β over IL-1 receptor antagonist production ratio have increased risk to develop MS than families with a low ratio (17).

IL-1β and IL-18 are produced as inactive precursors and require the cleavage by caspase-1 to become active (18). The activation of caspase-1 is mediated by the oligomerization of multi-protein complexes, known as inflammasomes (19, 20). To date, several inflammasomes have been described. Nonetheless, the NOD-like receptor protein 3 (NLRP3) is the most extensively studied.

The contribution of the NLRP3 inflammasome to human diseases has been demonstrated by studies revealing constitutively active forms of NLRP3 by mutations within the *Nlrp3* locus. The mutations correlate to autoinflammatory syndromes, such as Muckle-Wells syndrome, cryopyrin-associated periodic syndrome and familial cold autoinflammatory syndrome (21, 22). Indeed, NLRP3 inflammasome has been related to many human diseases, such as gout, type II diabetes and CNS diseases, such as MS (23–26). NLRP3 inflammasome also play a critical role in EAE pathogenesis since *Nlpr3*-deficient mice undergo milder EAE severity (27, 28). Similarly, administration of MCC950, a small NLRP3 inflammasome inhibitor, has shown efficacy in EAE mouse models (29, 30), suggesting the NLRP3 inflammasome as a new potential target for the treatment of MS.

OLT1177 (Dapansutrile) is a specific inhibitor of the NLRP3 inflammasome. OLT1177 is active *in vivo* and limits the severity of endotoxin-induced inflammation and joint arthritis (31). This drug was initially formulated as a candidate for the topical treatment of degenerative arthritis and subsequently the oral form was developed. Just as the topical gel, the oral capsules also are demonstrating that OLT1177 is safe and well-tolerated in humans (31, 32). In the current study, we assessed whether OLT1177 exerts therapeutic effects in a chronic model of EAE. We revealed that oral administration of OLT1177 mediated marked anti-inflammatory actions and ameliorated EAE severity in mice.

## Materials and Methods

### Experimental Autoimmune Encephalomyelitis

Female adult C57BL/6 (8–10 weeks old; Charles River Laboratories) were sedated with intramuscular injection of a mixture of ketamine (22 mg/kg) (Imalgen 1000, Merial) and xylazine (2.5 mg/kg) (Rompun, Bayer). EAE was actively induced by subcutaneous immunization with 300 μg of myelin oligodendrocyte glycoprotein peptide 35–55 (MOG_35−55_ MEVGWYRSPFSRVVHLYRNGK, Thermo Fisher Scientific, MA, USA) in 200 μl Complete Freund's Adjuvant (CFA) (Difco, MI, USA) supplemented with 4 mg/mL of heat inactivated *Mycobacterium tuberculosis* (Difco, MI, USA). Intraperitoneal (i.p.) injections of 400 ng of pertussis toxin (Sigma-Aldrich, ON, USA) in 100 μl sterile saline were also administered at the day of induction and again 48 h later. All the mice were housed with food and water *ad libitum* at a room temperature of 22 ± 2°C under 12:12 h light-dark cycle.

### Drug Administration

EAE-induced mice were randomly assigned to the OLT1177 treatment and control experimental groups. OLT1177 was administered orally or intraperitoneally.

#### Oral OLT1177 Administration

EAE-mice were fed either an OLT1177-enriched diet or standard food diet from the day same of the EAE induction. The composition of the food was identical, except that OLT1177-enriched food contained 3.75 g per kilogram of food. Standard and OLT1177-enriched food were prepared by Research Diets (New Brunswick, NJ, USA). Food and water were provided *ad libitum* for the entire length of the study, or 23 days post-EAE induction.

#### Intraperitoneal OLT1177 Administration

OLT1177 solubilized with sterile saline and administered i.p. daily until the end of the study (21 days). Two different administration protocols were tested: (i) 200 mg/kg OLT1177 injected once per day starting on the day of the EAE immunization; and (ii) 60 mg/kg OLT1177 injected twice a day starting on the day of the EAE induction. Control mice were administered saline on the same days.

#### Oral Gavage OLT1177 Administration

OLT1177 was solubilized with distilled water and administered daily (60 mg/kg), twice per day, by oral gavage. Treatment was initiated on the first day the animals displayed the first signs of EAE until the end of the follow up. Control mice were administered distilled water on the same days.

### Functional Evaluation

Mice were scored daily from day 0 to 21 or 23 after induction of EAE. The researcher was blind to the experimental groups during the functional evaluation. A 6-point scale was used to evaluate the clinical signs of EAE: 0 = normal walking, 0.5 = partially paralyzed tail, 1 = fully paralyzed tail, 2 = mild hind limb weakness, quick righting reflex, 3 = severe hind limb weakness, slow righting reflex, unable to bear weight, 3.5 = severe hind limb weakness and partial paralysis of hind limb, 4 = complete paralysis of at least one hind limb, 4.5 = complete paralysis of one or both hind limbs and trunk weakness, 5 = complete paralysis of one or both hind limbs, forelimb weakness or paralysis, 6 = mouse is found death by EAE.

### Histological Analysis

EAE mice were euthanised at either day 21 or 23 post-immunization with an overdose of pentobarbital sodium (Dolethal) and transcardially perfused with 4% paraformaldehyde (PFA) in 0.1 M phosphate buffer (PB). Lumbar segments of spinal cords were harvested, post-fixed in 4% PFA for 2 h and cryoprotected in 30% sucrose in 0.1 M at 4°C for at least 48 h. Spinal cords were embedded in TissueTek OCT (Sakura), cut in transversal sections (15 μm-thick) with a cryostat (Leica) between L3 and L5 segments and serially picked up on gelatine-coated glass slides. Samples were stored at −20°C.

Sections were stained with Luxol Fast Blue (LFB) (Sigma Aldrich). Briefly, after a graded dehydration, sections were placed in 1 mg/mL of LFB solution in 96% EtOH and 0.05% acetic acid overnight at 37°C and protected from light. Then, slides were washed with 96% EtOH, rehydrated in distilled water and placed in a 0.5 mg/mL Li_2_CO_3_ solution for 3–5 min at room temperature. Finally, sections were washed in distilled water, dehydrated again in 100% EtOH and mounted in DPX (Sigma Aldrich). To assess the demyelinated area in the spinal cord, 6 random images per mice were captured at 10× magnification with an Olympus BX51 and the attached Olympus DP73 Camera. The total demyelinated area within the spinal cord was measured with Image J image analysis software.

### Cytokine Protein Expression

EAE mice were euthanised 3 days after EAE onset with an overdose of pentobarbital sodium (Dolethal). Blood (500 μl) was obtained by cardiac puncture and centrifuged at 25,000 g for 10 min at room temperature to collect plasma separately from blood cells. Then, mice were transcardially perfused with 60 mL of sterile saline (0.9% NaCl). Spinal cords were harvested and rapidly frozen in liquid nitrogen. Protein isolation from the spinal cord samples and cytokine quantification was performed as we described previously (33). Protein levels of 6 cytokines (IL-1β, IL-18, IL-6, CXCL-1, TNFα, and IL-10) were analyzed using a custom-designed Cytokine Magnetic Bead Panel (Invitrogen) on a MAGPIX system (Millipore).

### Fluorescence Activated Cell Sorting (FACS)

Immune cell infiltration was determined in the spinal cord of EAE mice at disease peak. Mice were euthanised with an overdose of pentobarbital sodium (Dolethal) and transcardially perfused with 60 mL of sterile saline (0.9% NaCl). Spinal cords were collected, cut in small pieces and enzymatically dissociated in 1 mL of Hank's Balanced Salt Solution (HBSS) without Ca^2+^/Mg^2+^ containing 0.1% collagenase and 0.1% DNase for 30 min at 37°C. Then spinal cords were mechanically disintegrated by passing them with Dulbecco's Modified Eagle Medium (DMEM)-10% fetal bovine serum (FBS) through a 70 μm cell strainer to obtain a cell suspension (33).

Cell suspensions were split into different 1.5 mL microcentrifuge tubes according to the number of antibody combinations. Cells alone and isotype-matched control samples were generated to control for non-specific binding of antibodies and for autofluorescence. For extracellular staining, the following antibodies from eBioscience were used at a 1:300 concentration: CD45-PerCP, CD11b-PE or -PE-Cy7, F4/80-PE or -APC, Ly6C-FITC, Ly6G-PE, CD3-FITC-APC-PerCP; CD4-APC-Cy7, CD8-APC, CD49b-PE, CD24-PE. Samples were incubated with the primary antibodies for 1 h at 4°C in soft agitation, washed with DMEM-10% FBS, centrifuged twice at 300 g for 10 min at 4°C to remove debris and then fixed with 1% PFA. For intracellular staining, the following antibodies from eBioscience were also used at 1:300 concetration: FoxP3-PE-Cy7, tBet-PerCP, RORγ-APC, and GATA3-PE. After extracellular staining, cells were fixed and permeabilizated using FoxP3 Transcription Factor Staining Buffer Set (eBioscience) for 40 min at 4°C. Then, samples were immunostained with the intracellular antibodies over nigh at 4°C. Finally, stained cells were washed with PBS twice and fixed with 1% PFA.

Samples were analyzed on a FACS Canto Flow Cytometer (BD Bioscience) and all data were processed using FlowJo® software V.10.

### Statistical Analyses

Data are shown as mean ± standard error of the mean (SEM). The Kolmogorov-Smirnov test was used to check normality. Two tailed Student's *t*-test was used for the comparison between two different groups (histological analysis). One-way ANOVA was used for the comparison of two different doses and the control condition with *post-hoc* Bonferroni's test for multiple comparisons (cytokine assay, FACS analysis). EAE clinical score was analyzed by using two-way repeated measures ANOVA with *post-hoc* Bonferroni's test for multiple comparisons. Differences were considered significant at *p* < 0.05.

## Results

### OLT1177 Administration in Daily Diet Ameliorates Neurological Decline and Nervous Tissue Damage in EAE Mice

**Figure 1:**
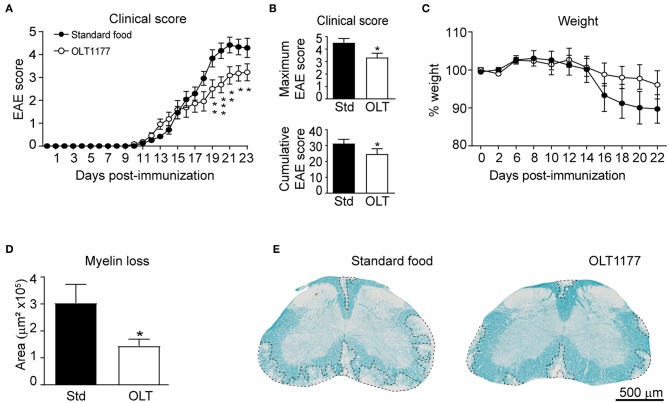
OLT1177-enriched food enhances functional and histological outcomes in EAE. **(A,B)** Graph showing the evolution of the EAE clinical score **(A)**, as well as, the cumulative and maximum EAE score **(B)** of mice fed a standard diet or a diet supplemented with 3.75 g per kg of food. **(C)** Plot showing the percent of weight of mice fed the standard and OLT1177-enriched food. **(D,E)** Graph showing the quantification of demyelination in the lumbar spinal cord of mice fed the standard food and 3.75 g/kg of OLT1177 at 23 days after induction **(D)** and representative spinal cord sections from each experimental group **(E)**. *p < 0.05; **p < 0.01; ***p < 0.001, 3.75 g/kg vs. Standard food. Two-way repeated measures ANOVA, Bonferroni's *post-hoc* test in **(A,C)** (*n* = 11 per group); Student's *t*-test in **(B)** (*n* = 11 per group); and **(D)** (*n* = 8 for Standard food and *n* = 11 for 3.75 g/kg of OLT1177). Data shown as mean ± sem.

We first aimed at investigating whether oral prophylactic treatment with OLT1177 led to beneficial effect in EAE. For this purpose, mice were fed a standard or OLT1177-enriched diet (3.75 g of OLT1177 per kg of food) from the day of the immunization until the end of the experiment. We found that OLT1177-enriched food ameliorated the neurological deficits of EAE disease (Figures 1A,B). We also observed that EAE mice fed the OLT1177 diet tended to show reduced weight loss, a feature associated with the disease progression. However, this effect was not statistically significant (Figure 1C).

Next, we studied whether OLT1177 protected against demyelination. In line with functional outcomes, histological analysis revealed that spinal cords from mice fed the lower OLT1177 dose diet had ~2-fold reduced demyelination than mice receiving the standard food (Figures 1D,E).

### OLT1177-Enriched Diet Modulates the Inflammatory Response in the Spinal Cord of EAE Mice

**Figure 2:**
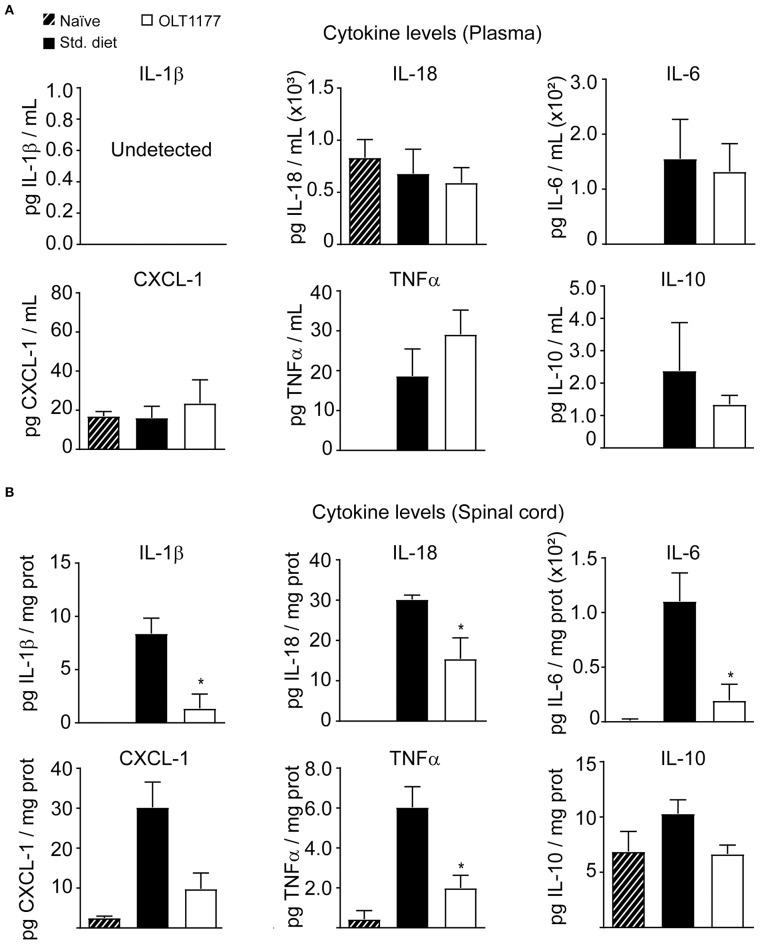
OLT1177-enriched food attenuates the protein levels of pro-inflammatory cytokines in the spinal cord of EAE mice at 3 days after the disease onset. **(A,B)** Protein levels of IL-1β, IL-18, IL-6, CXCL-1, TNFα, and IL-10 in the plasma **(A)** and in the spinal cord **(B)** in EAE mice fed standard food, or 3.75 g/kg OLT1177. *p < 0.05 vs. Standard food. One-way ANOVA, Bonferroni's *post-hoc* test (*n* = 4 per group). Data shown as mean ± sem.

We next sought to investigate whether OLT1177 attenuated inflammation in EAE mice. With this aim, we first studied whether mice fed the OLT1177-enriched food reduced cytokine levels in the blood plasma and spinal cord of EAE mice at 3 days after disease onset. In plasma these analyses revealed that OLT1177 did not change the concentration of any of the six cytokines assessed samples (Figure 2A). In the spinal cord, however, protein levels for IL-1β and IL-18 were significantly reduced in mice fed the OLT1177 diet compared with mice fed the standard diet (Figure 2B), corroborating the NLRP3 inhibition within the spinal cord. Moreover, the OLT1177-enriched food also reduced the levels of TNFα, CXCL-1 and IL-6 but did not affect the levels of the anti-inflammatory, IL-10 (Figure 2A). These data suggest that OLT1177 mediates anti-inflammatory actions in the CNS of EAE mice.

**Figure 3:**
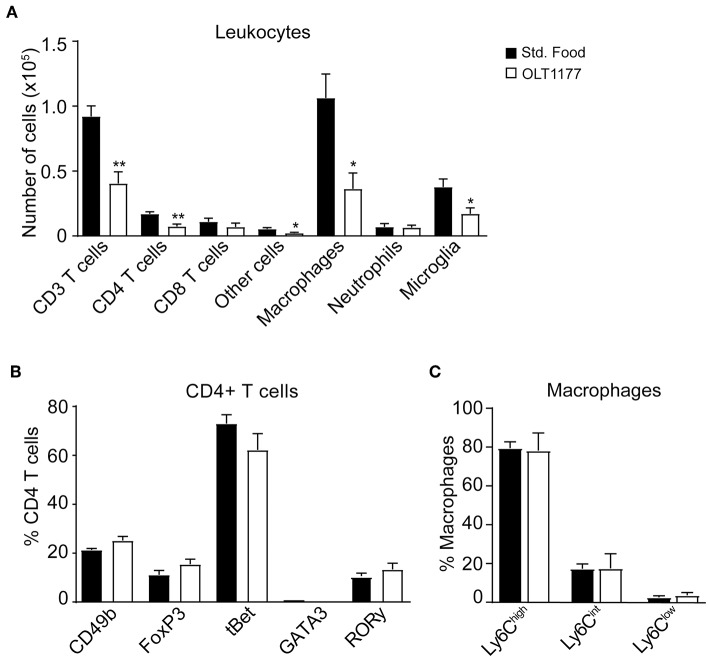
OLT1177-enriched food reduces the accumulation of immune cells in the spinal cord of mice at the peak of EAE. **(A–C)** Graphs showing the counts of different immune cell subsets in the spinal cord **(A)**, the percent of CD4^+^ T cells expressing the transcription factors CD49b, FoxP3, tBet, GATA3, or RORγ **(B)** and the percent of different macrophage subsets according to Ly6C expression **(C)** in the spinal cord of mice fed standard food and 3.75 g/kg OLT1177 at the peak of EAE. *p < 0.05; **p < 0.01 vs. Standard food. One-way ANOVA, Bonferroni's *post-hoc* test (*n* = 4 per group). Data shown as mean ± sem.

We then studied whether the reduction of cytokines in the spinal cord of EAE mice mediated by OLT1177 attenuated the accumulation of immune cells in the CNS at the peak of the disease or altered their phenotype. Flow cytometry analysis revealed that mice fed the OLT1177-enriched diet showed ~2-fold reduction in the infiltration of T cells (CD45^+^, CD3^+^), which was due to decreased counts of CD4 rather than CD8 T cells. The OLT1177-enriched diet also reduced the accumulation macrophages (CD45^high^, CD11b^+^, F4/80^+^), microglia (CD45^low^, CD11b^+^), and other cells (CD45^+^, CD11b^−^, CD3^−^, CD24^+^), but did not significantly decreased the counts of neutrophils (CD45^high^, CD11b^+^, F4/80^−^, Ly6G^+^) (Figure 3A, Supplementary Figures 1A–F). However, the OLT1177-enriched food did not lead to alteration in the polarization of CD4 T cells, as revealed by the expression of the transcription factors that identify Th1 (tBet), Th2 (GATA3), Th17 (RORγ) and the classical (FoxP3) and non-classical (CD49b) regulatory CD4 T cells (Figure 3B, Supplementary Figure 2A). Similarly, OLT1177 did not modify the proportion of pro-inflammatory (Ly6C^high^) and anti-inflammatory (Ly6C^low^) macrophages (Figure 3C, Supplementary Figure 2B).

### Effects of Therapeutic Administration of OLT1177 in EAE Mice

**Figure 4:**
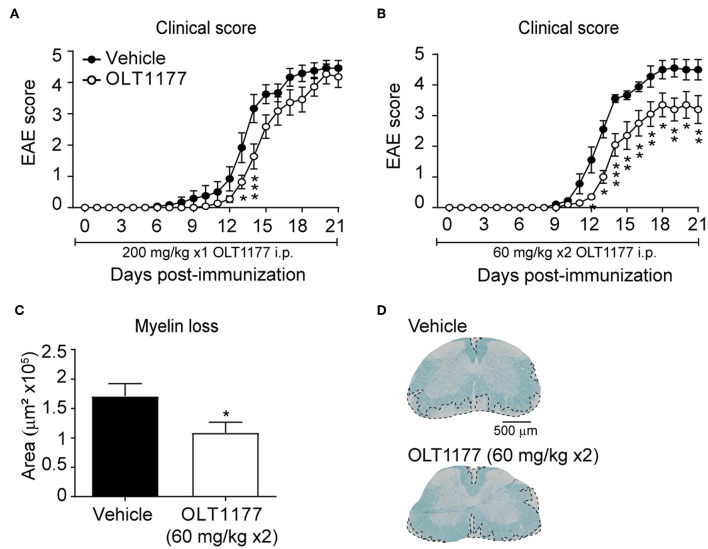
Effects of prophylactic treatment of OLT1177 EAE mice. **(A,B)** Average EAE clinical score of mice treated with single-daily i.p. administration of 200 mg/kg **(A)** or twice-daily administration of 60 mg/kg OLT1177 **(B)**. **(C,D)** Graph showing the quantification of demyelination in the lumbar spinal cord of mice treated with both OLT1177 doses **(C)** and representative images of lumbar spinal cord tissue sections stained against LFB from EAE treated with vehicle, single-daily 200 mg/kg and twice-daily 60 mg/kg of OLT1177 **(D)**. *p < 0.05; **p < 0.01; ***p < 0.001 vs. Vehicle. Two-way repeated measures ANOVA with Bonferroni's *post-hoc* test in **(A)** (*n* = 12 in vehicle and *n* = 10 in OLT1177) and **(B)** (*n* = 9 in vehicle and *n* = 10 in OLT1177); Unpaired *t*-test in **(C)** (*n* = 9 in vehicle and *n* = 10 in OLT1177). Data shown as mean ± sem.

We next sought to investigate whether OLT1177 mediates beneficial effects when administrated in EAE mice after disease onset. Since EAE mice undergo weight loss after disease onset due to reduced food intake, we aimed at delivering the OLT1177 by oral gavage once mice showed the first signs of the disease. For this purpose, and since the above described data reveal that OLT1177 losses efficacy at high doses, we first tested the effectivity of the prophylactic effects of two different OLT1177 administration regime in EAE: (i) single daily administration of OLT1177 (200 mg/kg mouse) from the day of the immunization; (ii) twice-daily administration of OLT1177 (60 mg/kg mouse) from the day of the induction. These experiments revealed that the single daily administration (200 mg/kg; i.p.) of OLT1177 did not prevent significantly neurological decline of EAE (Figure 4A), while the twice-daily dose of OLT1177 (60 mg/kg; i.p) ameliorated functional deficits and demyelination (Figures 4B–D).

**Figure 5:**
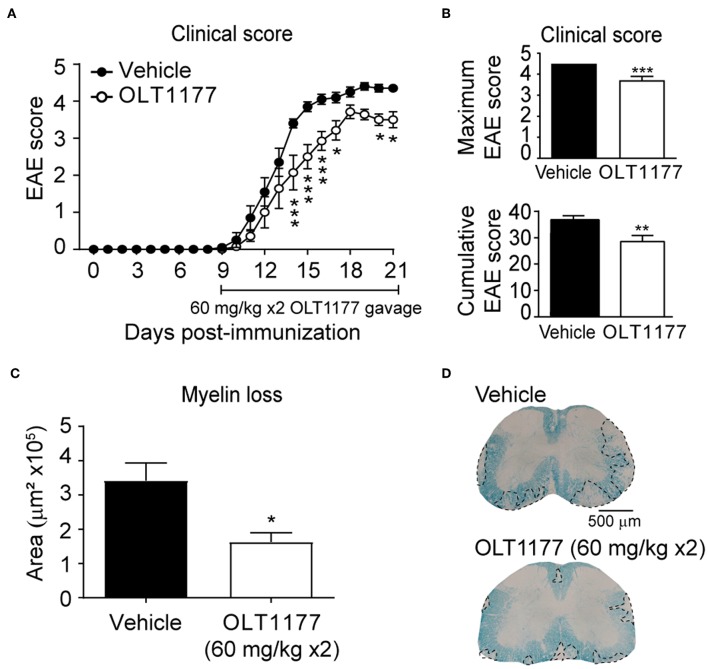
Oral administration of OLT1177 mediate therapeutic effects in EAE. **(A,B)** Plot showing the course of the EAE clinical **(A)** and cumulative and maximum clinical EAE score **(B)** of mice treated with twice-daily oral gavage of OLT1177 (60 mg/kg) or vehicle from disease onset. **(C)** Graph showing the quantification of demyelinating in the spinal cord of vehicle- or OLT1177-treated mice at 21 days post-induction. **(D)** Representative LFB stained lumbar spinal cord sections from OLT1177- and vehicle-treated mice at 21 days post-immunization. *p < 0.05; **p < 0.01; ***p < 0.001 vs. vehicle. Two-way repeated measures ANOVA with Bonferroni's *post-hoc* test in **(A)** (*n* = 10 for vehicle and *n* = 7 for OLT1177). Unpaired *t*-test in **(B)** (*n* = 10 for vehicle and *n* = 7 for OLT1177) and **(C)** (*n* = 9 in vehicle and *n* = 7 in OLT1177). Data shown as mean ± sem.

We then tested whether the twice-daily oral gavage (60 mg/kg mouse) of OLT1177, starting at disease onset, attenuated the clinical signs of EAE. We observed that mice treated with OLT1177 showed reduced neurological deficits despite treatment was initiated at disease onset. Indeed, mice treated with OLT1177 showed a reduction in ~1 point in the EAE score compared to mice given with vehicle (Figure 5A). Furthermore, OLT1177 also significantly reduced the cumulative and maximum clinical score in EAE (Figure 5B). In accordance to functional data, we also observed that OLT1177 protected against demyelination as revealed the histological analysis of LFB stained spinal cords (Figures 5C,D). These data support the beneficial effects of the therapeutic administration of OLT1177 in EAE.

## Discussion

MS is a highly disabling disease of the CNS that affects ~2.5 million people worldwide (2, 4, 34). There are currently some available therapies in the clinic for the treatment of MS. However, most of them show poor efficacy, especially in individuals who suffer from progressive MS. Moreover, they also have several side effects (35, 36).

Although the etiology of MS is still unknown, inflammation has a key implication in its physiopathology (2, 37). Pro-inflammatory cytokines, such as IL-1β or IL-18, have been extensively reported in mouse models to contribute to the pathogenesis of MS and EAE (9–13, 15, 16, 38). In this scenario, OLT1177 may emerge as an effective and safe candidate to treat this disease. OLT1177 is an active moiety discovered during the investigation of synthetic reactions containing chlorinating agents and methionine. OLT1177 inhibits the NLRP3 inflammasome, a macromolecular structure needed for the processing and release of IL-1β and IL-18, and has been shown in Phase I and II clinical trials to be safe when administered orally (and topically) in humans (18, 32).

In the current study, we reported that both oral and intraperitoneal OLT1177 administration exerted protective effects on functional and histological outcomes in EAE mice when given prophylactically. We uncovered that oral administration of OLT1177 to EAE mice decreased the concentration of pro-inflammatory cytokines in the spinal cord and the infiltration of immune cells. Importantly, therapeutic treatment of OLT1177 resulted in partial protection against neurological decline and demyelination when administered orally.

Previous reports revealed that OLT1177 inhibits specifically the NLRP3 inflammasome, and thus, prevents the autoproteolytic activation of caspase-1, needed for the processing and release of IL-1β and IL-18 (32). Caspase-1, together with IL-1β and IL-18, are increased in MS patients (9–12, 39). Pharmacological or genetic inhibition of either IL-1β or IL-18 protected against the pathogenicity of EAE (13–16, 38) suggesting an important role of NLRP3 in the pathogenesis of EAE. This is further supported by experiments using NLPR3-deficient mice, which displayed reduced EAE severity (27).

To our knowledge, there are two previous studies demonstrating that the pharmacological inhibition of the NLRP3 inflammasome, using a small molecule tool compound, MCC950, reduced functional impairments in a model of relapsing-remitting EAE when given prophylactically (29, 30). To this extent, we also showed the efficacy of the prophylactic inhibition of the NLRP3 inflammasome by OLT1177 in a model of chronic EAE, which is a more challenging condition.

A previous report demonstrated that mice fed an OLT1177-enriched diet reduced the clinical signs of arthritis (31). Here, we revealed that EAE mice fed an OLT1177-enriched food displayed enhanced functional and histopathological outcomes in a chronic model of EAE. These effects were likely due to the ability of this drug to reduce NLRP3 inflammasome activation, as revealed the lower protein levels of IL-1β and IL-18 in the spinal cord of EAE mice. We also found that OLT1177 in the diet led to marked attenuation of the levels of IL-6, a validated biomarker of IL-1β (40), CXCL-1 and TNFα in the spinal cord parenchyma of EAE mice. Importantly, OLT1177 did not reduced the protein levels of IL-10 in the spinal cord or blood of EAE mice, indicating that this drug is not altering the natural anti-inflammatory mechanisms to contain inflammation.

These data are consistent with the previous studies demonstrating the ability of this drug to reduce IL-1β and IL-6 levels in human monocyte-derived macrophages stimulated with LPS but also in animal models of arthritis (31, 32). Thus, OLT1177 seems to mediate its helpful effects by acting preferably in the CNS of EAE mice, since it did not alter cytokine concentrations in the blood. Previous studies revealed that monocytes and neutrophils are the main cell source of IL-1β in the spinal cord of EAE mice, and that IL-1β null mice, similar to NLRP3 knockout mice, are resistant to EAE (16). IL-1β expression in monocytes, but not in neutrophils, is important to allow the transmigration of proinflammatory monocytes across the blood–CNS barrier and to initiate neuroinflammation in EAE (41). IL-1β mediates these pathogenic effects in EAE by signaling via CNS endothelial cells IL-R1 to mediate GM-CSF production, which in turn, induces the differentiation of monocytes into antigen presenting cells within the perivascular space of CNS blood vessels (41). Moreover, myeloid cell-derived IL-1β also mediates direct activation of myelin-reactive T cells and stimulates the production of neurotoxic factors (41) which are likely to the be responsible, in part, of the CNS damage. Since OLT1177 suppressed ~80% the protein levels of IL-1β in the CNS of EAE mice, and infiltrating myeloid cells are the main cell source of IL-1β, it suggests that OLT1177 is likely to mediate its beneficial effects by targeting myeloid cell in the CNS. We also found that CD4 T cells and microglia cells were markedly reduced in the spinal cord by OLT1177, which is likely to be mediated as a consequence, in part, of IL-1β inhibition However, OLT1177-enriched diet did not altered the number of neutrophils at the time point analyzed (peak of EAE), contrary to previous reports on joint inflammation (31). Since neutrophils are recruited are very early stages of EAE and their counts are largely reduced after the peak of the disease, we do not discard that the lack of effects of OLT1177 in neutrophils accumulation in EAE could be due to the time assessed.

However, we cannot discard the possibility that this NLRP3 inhibitor could alter the activation of immune cells in the periphery despite the levels of circulating cytokines were not altered by OLT1177.

We also found that the administration of the low dose of OLT1177 twice per day showed greater efficacy in reducing EAE pathogenesis than the high dose once per day when given intraperitoneally. This effect may be explained due to the short half-life of OLT1177 within the organism. Importantly, we also showed that oral administration of OLT1177 reduced the clinical severity of EAE when treatment was initiated after disease onset. In these experiments, OLT1177 was administered by gavage and not directly in the diet because EAE mice show reduced food intake as a consequence of the disease. To our knowledge, this is the first study demonstrating the efficacy of therapeutic treatment in EAE with a selective NLRP3 inhibitor in clinical development, i.e., OLT1177. OLT1177 could have clinical relevance since it mimics a more suitable clinical scenario for MS patients. Importantly, oral OLT1177 has satisfactorily overcome a phase I safety trial in humans (32) and it is currently in a phase II clinical trials for acute gout flare (42), heart failure and rare disease which could be paving the way to clinical translation.

Overall, our data provide clear evidences that oral administration of OLT1177 exerts potent anti-inflammatory effects in EAE mice by inhibiting NLRP3 inflammasome and mediates beneficial effects when administered prophylactically and therapeutically in this model. This study therefore suggests that OLT1177 may constitute a novel safe and effective approach for the treatment of MS in humans.

## Data Availability Statement

The datasets generated for this study are available on request to the corresponding author.

## Ethics Statement

All experimental procedures were approved by the Universitat Autònoma de Barcelona Animal Experimentation Ethical Committee (CEEAH 2878) and followed the European Communities Council Directive 2010/63/EU, and the methods were carried out in accordance with the approved guidelines.

## Author Contributions

RL-V, DS, and CD designed the study. AS-F and RL-V performed the research, analyzed or interpreted the results. AS-F, RL-V, DS, and CD wrote the manuscript. All authors read and approved the final manuscript.

### Conflict of Interest

CD serves as Chairman of Olatec's Scientific Advisory Board, is co-Chief Scientific Officer, and receives compensation. DS serves as Chairman and Chief Executive Officer of Olatec. The authors declare that this study received funding from Olatec Therapeutics LLC. The funder had the following involvement with the study: provided the OLT1177 and contributed to the design of the study and the preparation of the manuscript.

## Supplementary Material

Supplementary Figure 1 OLT1177 enriched food reduces the accumulation of immune cells in the spinal cord of mice at the peak of EAE. **(A–F)** Representative dot plots showing neutrophils **(A)**, macrophages and microglia **(B)**, T lymphocytes **(C)**, Other cells **(D)**, T helper cells **(E)**, and T cytotoxic cells **(F)** in the spinal cord of mice fed the standard food or OLT117 enriched diet.

Supplementary Figure 2 OLT1177 enriched food did not modify the phenotype of lymphocytes nor macrophages in the spinal cord of EAE mice at the peak of EAE. **(A)** Representative flow cytometry histograms characterizing the expression CD4 T cells polarization markers in the spinal cord of mice fed the standard food or OLT117 enriched diet at EAE disease peak. **(B)** Representative dot plots showing different macrophage subsets in the spinal cord from the same animals based on the expression of Ly6C.
