# Supplementary figures and images for: OLT1177 (Dapansutrile), a Selective NLRP3 Inflammasome Inhibitor, Ameliorates Experimental Autoimmune Encephalomyelitis Pathogenesis
Source: Front Immunol. 2019 Nov 1;10:2578. doi: 10.3389/fimmu.2019.02578 (PMC6839275; doi:10.3389/fimmu.2019.02578)

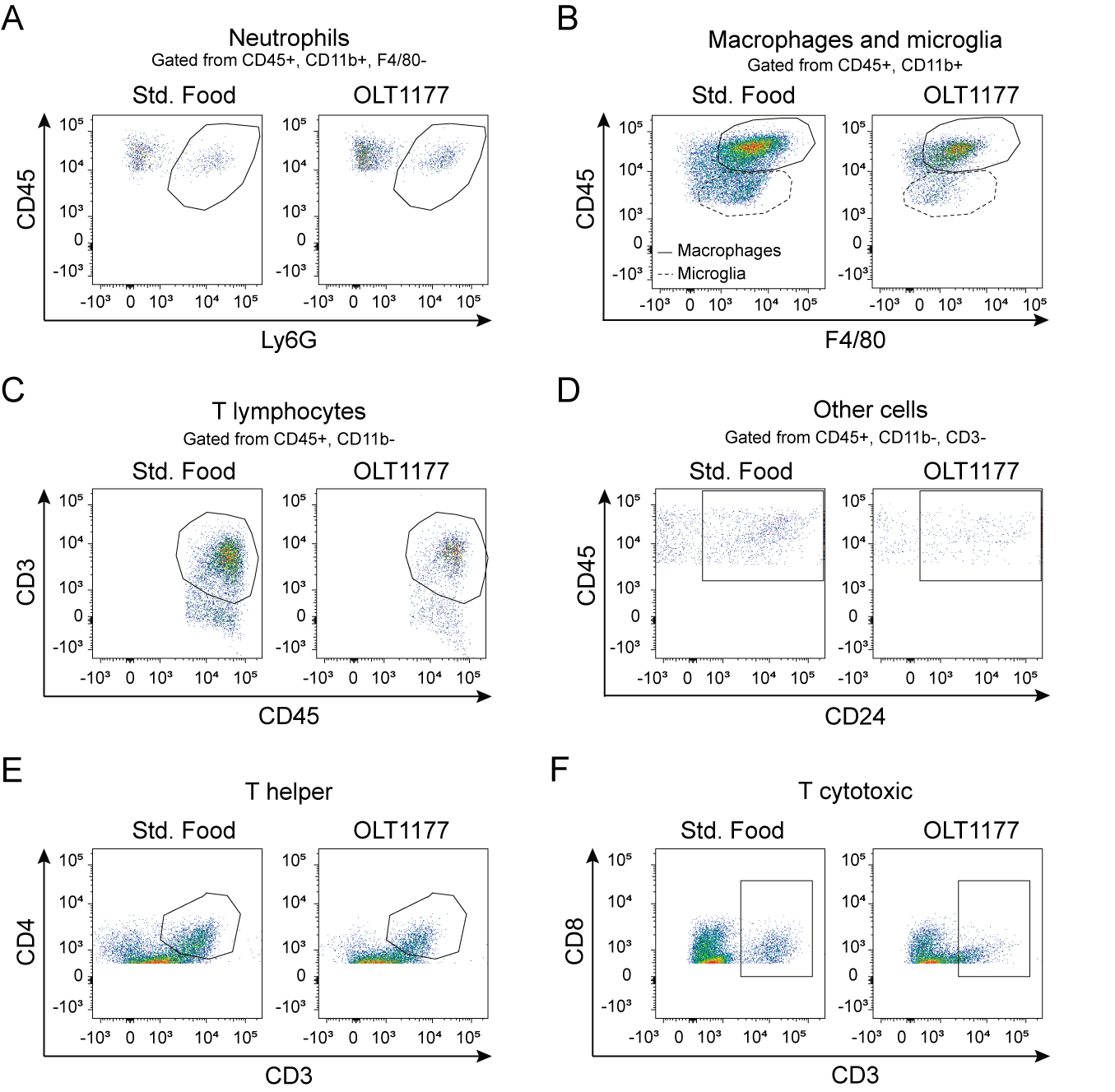

Supplement: Supplementary Figure 1 — OLT1177 enriched food reduces the accumulation of immune cells in the spinal cord of mice at the peak of EAE. (A–F) Representative dot plots showing neutrophils (A), macrophages and microglia (B), T lymphocytes (C), Other cells (D), T helper cells (E), and T cytotoxic cells (F) in the spinal cord of mice fed the standard food or OLT117 enriched diet. [file Image_1.TIF]

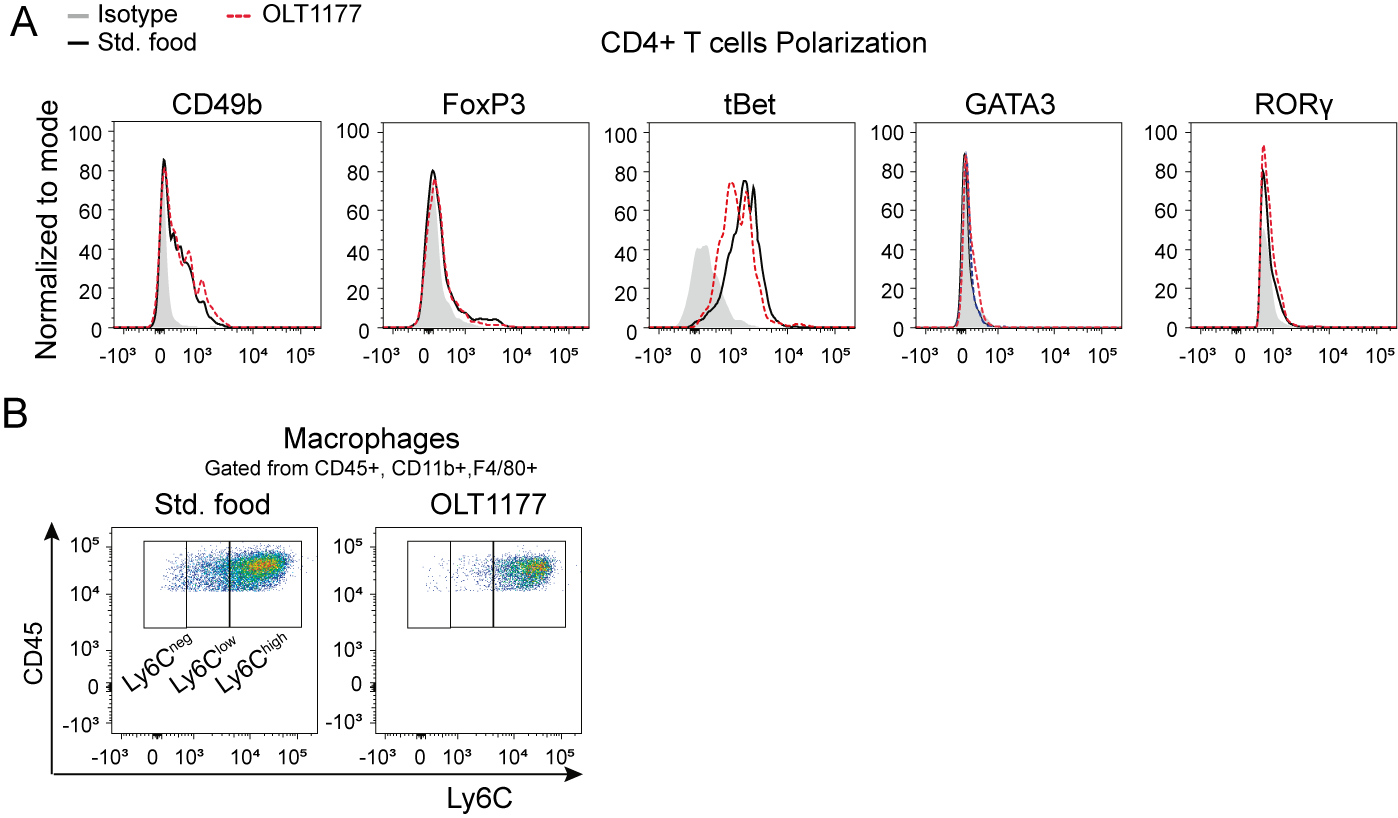

Supplement: Supplementary Figure 2 — OLT1177 enriched food did not modify the phenotype of lymphocytes nor macrophages in the spinal cord of EAE mice at the peak of EAE. (A) Representative flow cytometry histograms characterizing the expression CD4 T cells polarization markers in the spinal cord of mice fed the standard food or OLT117 enriched diet at EAE disease peak. (B) Representative dot plots showing different macrophage subsets in the spinal cord from the same animals based on the expression of Ly6C. [file Image_2.TIF]
